# Supplementary material for: iCLOTS: open-source, artificial intelligence-enabled software for analyses of blood cells in microfluidic and microscopy-based assays
Source: Nat Commun. 2023 Aug 18;14:5022. doi: 10.1038/s41467-023-40522-4 (PMC10439163; doi:10.1038/s41467-023-40522-4)
Supplement: Supplementary file 1 — Supplementary Information [file 41467_2023_40522_MOESM1_ESM.pdf]

Supplementary Information

iCLOTS: open-source, artificial intelligence-enabled software for analyses of blood cells in microfluidic and microscopy-based assays

Meredith E. Fay[1,2,3,4,5], Oluwamayokun Oshinowo[1,2,3,4,5], Elizabeth Iffrig[1,6], Kirby S. Fibben[1,2,3,4,5], Christina Caruso[2], Scott Hansen[7], Jamie O. Musick[2], José M. Valdez[7], Sally Azer[1,2,5], Robert G. Mannino[1,2,3,4,5], Hyoann Choi[1,2,3,4,5], Dan Y. Zhang[4,8], Evelyn K. Williams[1,2,3,4,5], Erica N. Evans[2], Celeste K. Kanne[2], Melissa L. Kemp[1,3,4], Vivien A. Sheehan[2], Marcus A. Carden[9], Carolyn M. Bennett[2], David K. Wood[7], Wilbur A. Lam[1,2,3,4,5,*]

[1] The Wallace H. Coulter Department of Biomedical Engineering, Georgia Institute of Technology &Emory University, Atlanta, GA, USA
[2] Department of Pediatrics, Division of Pediatric Hematology/Oncology, Aflac Cancer Center and Blood Disorders Service of Children's Healthcare of Atlanta, Emory University School of Medicine, Atlanta, GA, USA
[3] Winship Cancer Institute of Emory University, Atlanta, GA, USA
[4] Parker H. Petit Institute of Bioengineering and Bioscience, Georgia Institute of Technology, Atlanta, GA, USA
[5] Institute for Electronics and Nanotechnology, Georgia Institute of Technology, Atlanta, GA, USA
[6] Department of Medicine, Division of Pulmonary, Allergy, Critical Care, and Sleep Medicine, Emory University, Atlanta, GA, USA
[7] Department of Biomedical Engineering, University of Minnesota, Minneapolis, MN, USA
[8] The George W. Woodruff School of Mechanical Engineering, Georgia Institute of Technology, Atlanta, GA, USA
[9] Department of Epidemiology, Gillings School of Public Health, University of North Carolina, Chapel Hill, NC, USA

Supplementary methods

Single cell tracking workflows

Deformability assay microfluidic device preparation

Microchannels (5.9 ± 0.08 µm wide, 200 µm long) within the previously described cell deformability microfluidic device[1] were incubated with 0.2% (g/mL) bovine serum albumin (BSA; Sigma) in PBS and incubated for one hour at room temperature to coat the exterior and minimize interactions between the device wall and blood components. A microchannel height of 5 µm was prepared for RBCs and reticulocytes and a microchannel height of 13.8 µm was prepared for WBCs and cell lines.

Brightfield red blood cell deformability assay

Patients with sickle cell disease (SCD) had 2.7 mL of whole blood collected via venous blood draw in sodium citrate phlebotomy tubes. All SCD patients had a Hemoglobin SS (HbSS) genotype and had not received a blood transfusion in over 90 days prior to blood draw. Samples of blood from healthy adult volunteers were obtained via venous blood draw in sodium citrate tubes on the same day as SCD patient samples to be used for time-matched controls. Individuals undergoing laboratory evaluation who met criteria for iron deficiency anemia (IDA) (defined as hemoglobin below the normal reference range for age and ferritin < 10ng/mL) had 1mL of whole blood collected via venous blood draw in EDTA phlebotomy tubes. Samples of blood from healthy adult volunteers were obtained via venous blood draw in EDTA tubes on the same day as IDA patient samples to be used for time-matched controls. Following collection of all samples blood was stored at 4ºC until the time of experiments. To isolate RBCs from other blood components, blood samples were first centrifuged under a low acceleration of 150g for 15 minutes, washed with PBS, and centrifuged at 201g for 10 minutes. An additional wash and centrifugation were done to ensure complete isolation of RBCs.

Isolated control RBCs, SCD RBCs, or IDA RBCs (idRBCs) were diluted in PBS and perfused into the cell deformability microfluidic devices at a rate of 1 µL/min using a syringe pump (Harvard Apparatus). Samples for experiments with idRBCS were diluted to a ratio of 1:100 volume cells:PBS and SCD RBCs were diluted to a ratio of 1:200 volume cells:PBS to minimize cell clumping. Videomicroscopy was acquired at a rate of 25 FPS (20x, Nikon Eclipse TE2000-U).

Brightfield white cell line deformability assay

Acute T-cell lymphoblastic (Jurkat) cell line was grown and maintained using Roswell Park Memorial Institute (RPMI) 1640 Medium (Lonza), 10% fetal bovine serum (Rockland Immunochemicals) and 1% penicillin-streptomycin (Gibco). Acute promyelocytic leukemia (HL-60) was grown and maintained using Iscove's Modified Dulbecco's Medium (IMDM) (Gibco), 20% fetal bovine serum (Rockland Immunochemicals) and 1% penicillin-streptomycin (Gibco). Cells were suspended in PBS at a concentration of 1 M/mL and

perfused into the cell deformability microfluidic devices at a rate of 2.5 µL/min using a syringe pump (Harvard Apparatus). Videomicroscopy was acquired at a rate of 25 FPS (20x, Nikon Eclipse TE2000-U).

Fluorescent CD71+ reticulocyte deformability assay

Reticulocytes were isolated using a series of gradient-based methods[2]. After Percoll (Sigma) separation, PBS was aspirated and resultant reticulocyte pellet was resuspended in 1mL of chilled (4°C) PBS. 1:100 CD71+ anti-human antibody (Miltenyi Biotec, cat. # 130-098-779) was added and the sample was incubated for 15 minutes at room temperature. 1:500 Alexa Fluor-568 goat anti-mouse secondary antibody (Invitrogen, cat. # A-11004) was then added and sample was perfused into the cell deformability microfluidic devices at a rate of 1 µL/min using a syringe pump (Harvard Apparatus). Videomicroscopy was acquired at a rate of 25 FPS (20x, Nikon Eclipse TE2000-U).

Single cell tracking computational analysis methods

Single cell velocity (v) is calculated from data points meeting quality standards (equation 1).

$$v = (x_f - x_i) * FPS \tag{1}$$

where x_f and x_i represent initial and final x-position detection of an individual cell within subsequent frames. Frames per second (FPS) is the rate of video acquisition and is provided by the software user as an input. Current iCLOTS version 0.1.1 reports velocity per cell as the average calculated from all frames the cell is detected. A specialized x-direction channel flow single cell tracking application, which was used for deformability velocity measurements, is offered (equation 2).

$$v = \frac{x_f - x_i}{n_{frame,f} - n_{frame,i}} * FPS \tag{2}$$

where x_f and x_i represent initial and final x-position of an individual cell across all detected frames and $n_{frame,i}$ and $n_{frame,fl}$ indicate the first and last video frames an individual cell is detected, respectively. Average fluorescence intensity of individual cells was calculated by taking the mean sum of pixel intensity of the indicated cell region from all frames the cell was detected.

Cell suspension velocity workflows

Deoxygenation cell suspension velocity assay

SCD whole blood sample for the rheological measurement was collected at Children's Minnesota Hospital in Minneapolis, MN under approved protocol by the Institutional Review Boards at the University of Minnesota and Children's Minnesota. The sample was stored at 4°C in a 4 mL citrate tube after collection. A 200 µL aliquot of the sample was removed and centrifuged for 5 minutes at 300g two times. After each centrifugation, the supernatant was removed and replaced with Dulbecco's phosphate buffered saline (DPBS). After a final centrifugation for 10 minutes at 300g, all the buffer was removed and the red cells were added to a new aliquot to achieve a final RBC hematocrit of ~25% in DPBS.

Cell suspension in sepsis velocity assay

Patient samples were collected from a cohort identified within 48 hours of admission to a single ICU who were assigned a diagnosis of sepsis as defined by Sepsis-III[3] criteria – clinical suspicion or confirmation of infection with a change in the Sequential Organ Failure Assessment (SOFA) score of at least two. All samples were collected in tubes coated with EDTA as an anticoagulant and analyzed within 4 hours of collection. Control samples were obtained in the outpatient setting from nominally healthy volunteers free of infection in a similar manner and used within the same time frame. A microfluidic device with a straight channel portion (10 µm tall, 70 µm wide) was incubated with 1% (g/mL) BSA in PBS to coat the exterior and minimize interactions between the device wall and blood components. Ten second videos were recorded for each conditions with frame resolution of 1200x1920 pixels at 20x magnification at a rate of 160 FPS with minimal file compression.

KLT velocity tracking computational analysis methods

Velocity of each particle is calculated (equation 3).

$$v = \sqrt{(x_{n+1} - x_n)^2 + (y_{n+1} - y_n)^2} * FPS \tag{3}$$

Where ν indicates velocity, x, y indicate position of a detected feature over subsequent frames, and FPS indicates the frame rate of the video analyzed.

Cell adhesion workflows

Platelet isolation for all assays

Blood was drawn into acid-citrate-dextrose solution (ACD; VWR). The sample was subsequently centrifuged at 150g for 15 min without brake and the resultant platelet rich plasma (PRP) was centrifuged again with an additional 10% ACD by volume at 900g for 5 min without brake. The supernatant was discarded and the platelet pellet was resuspended into HEPES (VWR) modified Tyrode's buffer.

Brightfield microscopy platelet adhesion assay

Platelets were diluted to a concentration of 20 M/mL in Tyrode's buffer.

Brightfield microscopy red blood cell adhesion assay

Microfluidic devices with four separate channels (46 µm tall, 100 µm wide, 4 mm long) were coated with 10 µg/mL of laminin derived from human placenta (Sigma) for 2 hours at room temperature. After incubation, channels were washed with PBS (VWR). Whole blood collected in ethylenediamine tetraacetic acid (EDTA) solution (VWR) was washed 3 times at 200g for 15 minutes with PBS to isolate RBCs. RBCs were resuspended in PBS to 0.2% hematocrit. Devices were perfused with RBC suspension via syringe pump (Harvard Apparatus) at 0.3µL/min for 25 minutes. Images were acquired using a Keyence BZ-X810 microscope with a 20x/0.8 objective.

Brightfield microscopy adhesion computational analysis methods

Individual cells are located as particles using Trackpy as described for previous iCLOTS methods. Calculated values for eccentricity and radius of gyration of each cell particle are reported as circularity and radius, respectively. Radius is converted to area using formula $A = \pi r^2$. Radius of gyration is approximately equal to radius for red blood cells and platelets in brightfield microscopy images.

Neutrophil isolation for all assays

Neutrophils were isolated from whole blood collected in EDTA using a whole blood human neutrophil isolation kit (Miltenyi MACSxpress) and residual RBCs were lysed with an RBC lysis solution (Miltenyi) per manufacturer's instructions. Neutrophil suspension was centrifuged at 200g for 5 minutes and the pellet was reconstituted in 500 µL PBS.

Transient neutrophil adhesion assay

A straight channel microfluidic device (38 µm tall, 100 µm wide) was incubated with fibronectin (Sigma) at a concentration of 500 µg/mL at room temperature to coat the interior of the channel. The device was then washed with 1% (g/mL) BSA (Sigma) in PBS and incubated for at least 15 minutes at room temperature to coat the exterior and minimize interactions between the device wall and blood components. 35 µL of 0.1 M N-Formylmethionyl-leucyl-phenylalanine (fMLP; Sigma) was added to neutrophil solution, which was then perfused at a rate of 0.09 µL/min through the microfluidic device. Videomicroscopy was acquired at a rate of 25 FPS (10x, Nikon Eclipse TE2000-U).

Transient adhesion cell tracking computational analysis methods

Transit time is calculated (equation 4).

$$transit\ time = \left(n_{frame,f} - n_{frame,i}\right) * FPS \tag{4}$$

$n_{frame,i}$ and $n_{frame,fl}$ indicate the first and last video frames an individual cell is detected, respectively. Frames per second (FPS) is the rate of video acquisition and is provided by the software user as an input.

Fluorescence microscopy neutrophil adhesion assay

Isolated neutrophils were suspended in PBS at a concentration of 2×10^6 cells/mL and incubated with 10 ug/mL CellMask Deep Red plasma membrane stain (Invitrogen) and 5 μM SYTO 13 Green nucleic acid stain (Invitrogen) for 15 minutes at 37°C. The stained cells were washed and fixed with 4% paraformaldehyde (Sigma) for 10 minutes at room temperature. The cells were added to a non-coated glass chamber and stored at 4°C.

Fluorescence microscopy platelet adhesion assay

Platelets were diluted to 20 M/mL to ensure the measurement of single platelets and not platelet aggregates and were incubated on coverslips[4]. Platelets were allowed to adhere for 2 hours at room temperature.

Multiscale microfluidic accumulation workflows

Commercial microfluidic device occlusion and accumulation assay

Ibidi 0.2mm μ-Slide I Luer with ibitreat devices were filled with 100 μg/mL collagen IV (VWR) and incubated at room temperature for 30 minutes. In order to facilitate perfusion, syringes were loaded with 1 mL of whole blood treated with 40 μg/ml corn trypsin inhibitor (CTI; Haematologic Technologies), 1:100 Anti-CD45 Mouse Monoclonal Antibody (VWR, clone HI30, cat. # 304002-BL), 1:200 Integrin alpha 2b/CD41 Antibody (VWR, cat. # 10088-996), and 6mM $CaCl_2$. Blood was perfused using constant flow via a syringe pump (Harvard Apparatus) into devices for a period 10 minutes.

Microvasculature-on-a-chip device occlusion and accumulation assay

Endothelialized branching microfluidic devices with 32 microchannels (30 μm wide, 30 μm tall, 200 μm long) were prepared by growing a confluent layer of human umbilical vein endothelial cells (HUVECs, Lonza, cat. # cc-2519) on the surfaces of the channels of the microfluidic device[5]. Once devices reached endothelial cell confluency, typically 48 hours after seeding, CellMask Deep Red Plasma Membrane Stain (Fisher) was added to the culture media to pretreat devices for 20 minutes. In order to facilitate perfusion, syringes were loaded with 1 mL of whole blood treated with 40 μg/mL CTI, 1:100 Anti-CD45 Mouse Monoclonal Antibody (VWR), 1:200 Integrin alpha 2b/CD41 Antibody (VWR), and 3mM $CaCl_2$. Blood was perfused using constant flow via a syringe pump (PhD Ultra, Harvard Apparatus) into devices for 28 minutes. In branching microfluidic devices such as the device presented here, flow distribution may change over time due to the heterogenous distribution of accumulation.

Occlusion and accumulation computational analysis methods

Percent occlusion of a full device or an individual microchannel is calculated (equation 5).

$$O = \frac{a_{fl}}{a_{channel}} * 100 \tag{5}$$

O indicates percent occlusion, a_{fl} indicates summed pixel intensity values of fluorescence microscopy signal above the set threshold from a region of interest from the binarized image, and $a_{channel}$ indicates the area of the region of interest representing the channel. Accumulation from sequential images is calculated (equation 6).

$$A = \frac{a_n - a_{n-1}}{t} \tag{6}$$

A indicates accumulation, a_n and a_{n-1} indicates area of signal from a frame and the previous frame, and t indicates the time elapsed between measurements.

Software access

The current version of the iCLOTS software for Windows and Mac operating systems is available for download at the dedicated software website, https://www.iCLOTS.org, and all source code is available at https://www.github.com/iCLOTS. iCLOTS contains detailed documentation available at on the iCLOTS website or accessible within the software in dedicated help windows. Sample data, including sample analysis, is available for all applications at https://www.iCLOTS.org/software. All data presented and detailed experimental protocols are available upon request. For users with computational experience, all methods are also available as scripts independent of the standalone software at https://www.github.com/LamLabEmory. iCLOTS is under continuous development and as such the authors welcome all requests for assistance, questions, concerns, ideas, and feedback. Please contact us via the corresponding author, via the contact form on the iCLOTS website, or via the contact information available at https://www.github.com/LamLabEmory or https://www.github.com/iCLOTS.

Software use

iCLOTS has been designed as a standalone software to reach the widest range of users possible and for potential use in clinical environments. As such, no supporting software or software dependencies are required. No additional resources are needed to run the program. iCLOTS is installed simply by downloading the appropriate files. On Mac OS, users click the tar.gz distribution file to open, then click the .app file to start the software. On windows, users can click on the .exe file directly. iCLOTS initial version v0.1.1 is approximately 150 MB large, so may take 1-10 minutes to download, depending on

internet speed. iCLOTS will take an additional 1-5 minutes to open, particularly for first time use. The development team has taken the necessary steps to identify ourselves as legitimate developers to Mac and Windows OS. Upon opening for the first time, you may receive messages alerting you that the software has been downloaded from the internet and/or that iCLOTS is a new piece of software. The source of the software will be attributed to Meredith Fay, lead developer. During testing, all software users received and accepted these messages with no negative effect to their computers.

iCLOTS requires a 64-bit operating system and a minimum of 8 GB RAM, with more suggested for large .avi file video sets. Typically, after users adjust necessary parameters, analyses take on the order of seconds to minutes to run. To reduce processing time, users may resize or shorten files using the suite of video processing tools.

Application	Smaller dataset		Larger dataset	
Fluorescence microscopy adhesion	One 60x 62 KB image	<1 second	20 60x images totaling 1.6 MB	8 seconds
Fluorescence microscopy protrusion count	One 60x 62 KB image	<1 second	20 60x images totaling 1.6 MB	10 seconds
Single cell tracking	5.1 MB video with ROI selected	13 seconds	1.8 GB movie with ROI selected	1 minute, 33 seconds
Velocity profile	900 KB video	1 minute, 53 seconds	386 MB movie with ROI selected	2 minutes, 19 seconds
Microchannel accumulation	One tile scan 1.6 MB image	<1 second	9 tile scan images totaling 13.9 MB	2 seconds

Supplementary table 1. **iCLOTS analysis run times depending on application chosen and file size.** After the user takes time to adjust parameters to their own specific dataset, analysis of the dataset takes on the order of seconds to minutes, with larger video files requiring the most time. Parameters and ROI chosen may affect analysis time. Exporting data may take additional time, particularly if a large number of labeled video frames are being exported. Tests were performed on a 2019 MacBook Pro with 16 GB RAM. Individual processing times may vary based on computer model and specifications.

Supplementary figures

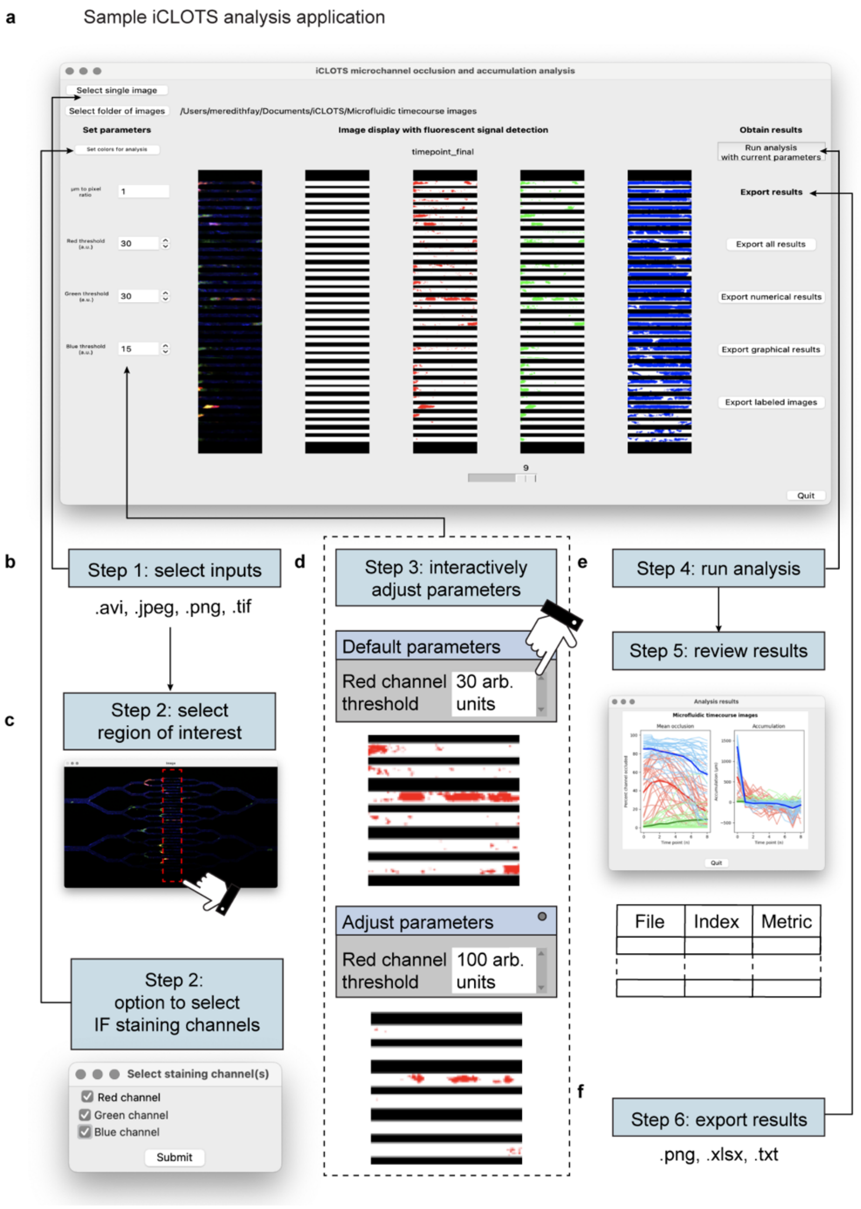

Supplementary figure 1. **Microfluidic-based applications within iCLOTS software easily adapt to variable channel dimensions.** All iCLOTS applications follow the same common interactive format, shown here with the microscale microfluidic accumulation workflow. **a** Upon selection of desired application, the analysis window is opened. **b** Software accepts common-format single or multiple image or video files as inputs. Here, a time course series of images is presented and can be accessed through a scroll bar beneath the analysis images. Users may scroll through frames using <Left> and <Right> arrow keys. **c** Depending on application and file type, software users are guided through a series of windows describing their data, such as indicating multiple immunofluorescence

staining channels present in a file. **d** Threshold parameters can be adjusted interactively from a default value to best match the pixel intensity within a software user's unique data set. **e** After parameters are selected, the software user indicates that the final analysis should be run. Upon completion, graphical results for each file are automatically displayed. **f** Numerical and graphical results with the corresponding labeled images are exported in easy-to-interpret formats.

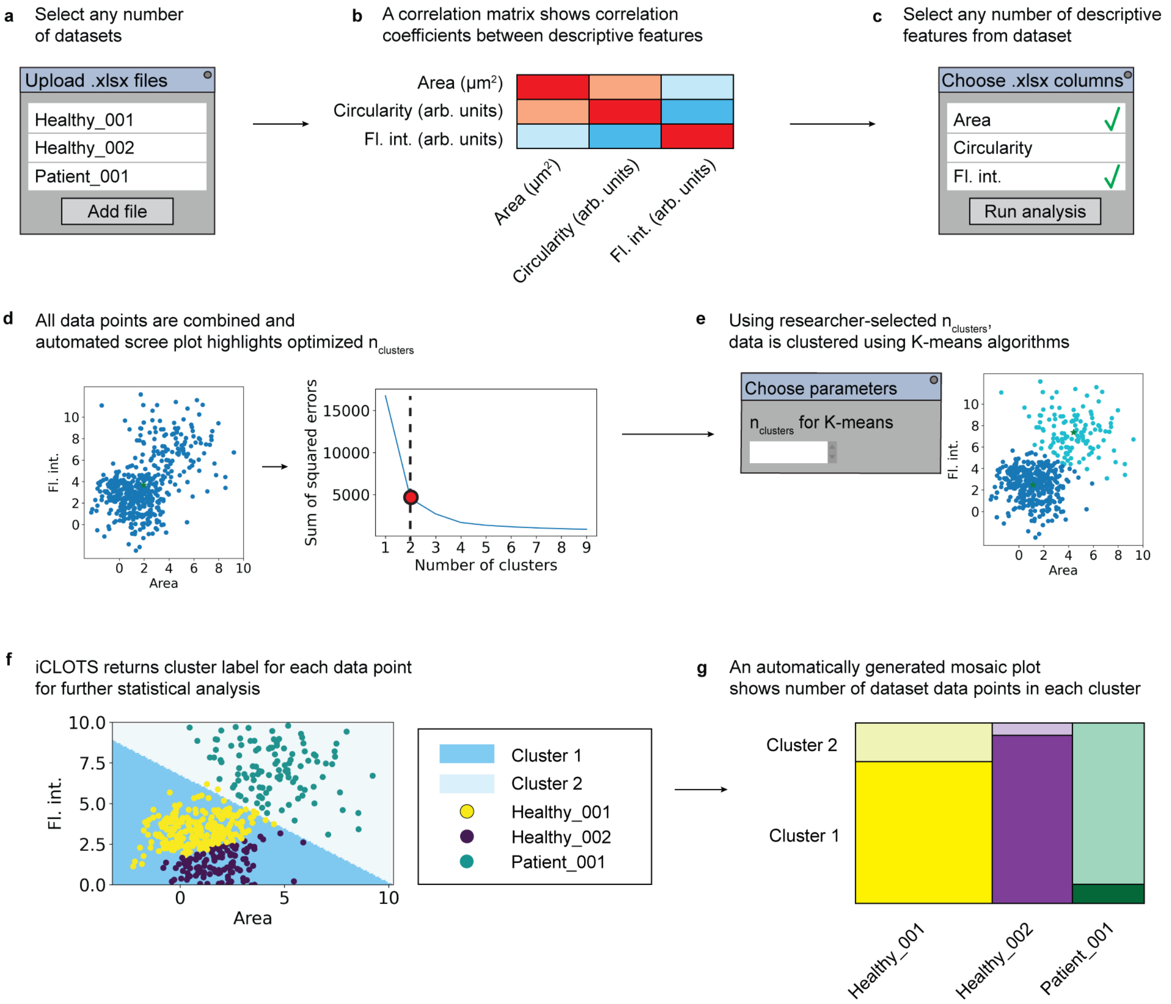

Supplementary figure 2. **iCLOTS software applies machine learning algorithms to generated data as a post-processing interpretation step.** Synthetic data and example graphical representations are used here to best demonstrate iCLOTS ML workflow and capabilities. Two-dimensional (tabular data with each cell described by two metrics) sample data is used to best visualize clustering capabilities, but iCLOTS ML application can accommodate any number of dimensions within a dataset. **a** The software user can upload any number of .xlsx files as algorithm inputs. Each .xlsx file is considered one dataset. **b** From these datasets, iCLOTS detects any columns common to all .xslx files uploaded. iCLOTS automatically generates a correlation matrix to indicate potential redundancy in all provided descriptive features. **c** Users can select any number of columns to be used in ML clustering. **d** Data points from all data sets are combined and a scree plot analysis is performed to indicate a suggested optimal number of mathematically significant clusters to retain. A scree plot is a line plot of the sum of squared errors (SSE) of the distance to the closest centroid for all data points for each number of clusters. Typically, as the number of clusters increases, the variance, or sum of squares, for each cluster group decreases. The indicated “elbow” point represents the best balance between minimizing the number of clusters and minimizing the variance in

each cluster. **e** The user can choose any number(s) of clusters to group data into using a k-means algorithm. Clustering algorithms find data points that have similar numerical values. k-means clustering is a method of vector quantization designed to partition data points into k clusters in which each point belongs to the cluster with the nearest mean. **f** iCLOTS calculates mathematically defined clusters and returns a cluster label for each data point provided. These labels can be used for further statistical analysis. **g** A mosaic plot, a specialized stacked bar chart that shows the number data points from each dataset in each of the clusters, is displayed to assist the user in visualizing the contribution of each dataset to each cluster.

a Comparison of manual analysis and iCLOTS results

Analysis method	Analysis time, single video
iCLOTS	< 2 min
Manual, participant 1	2 hours
Manual, participant 2	2 hours
Manual, participant 3	1 hour, 40 min

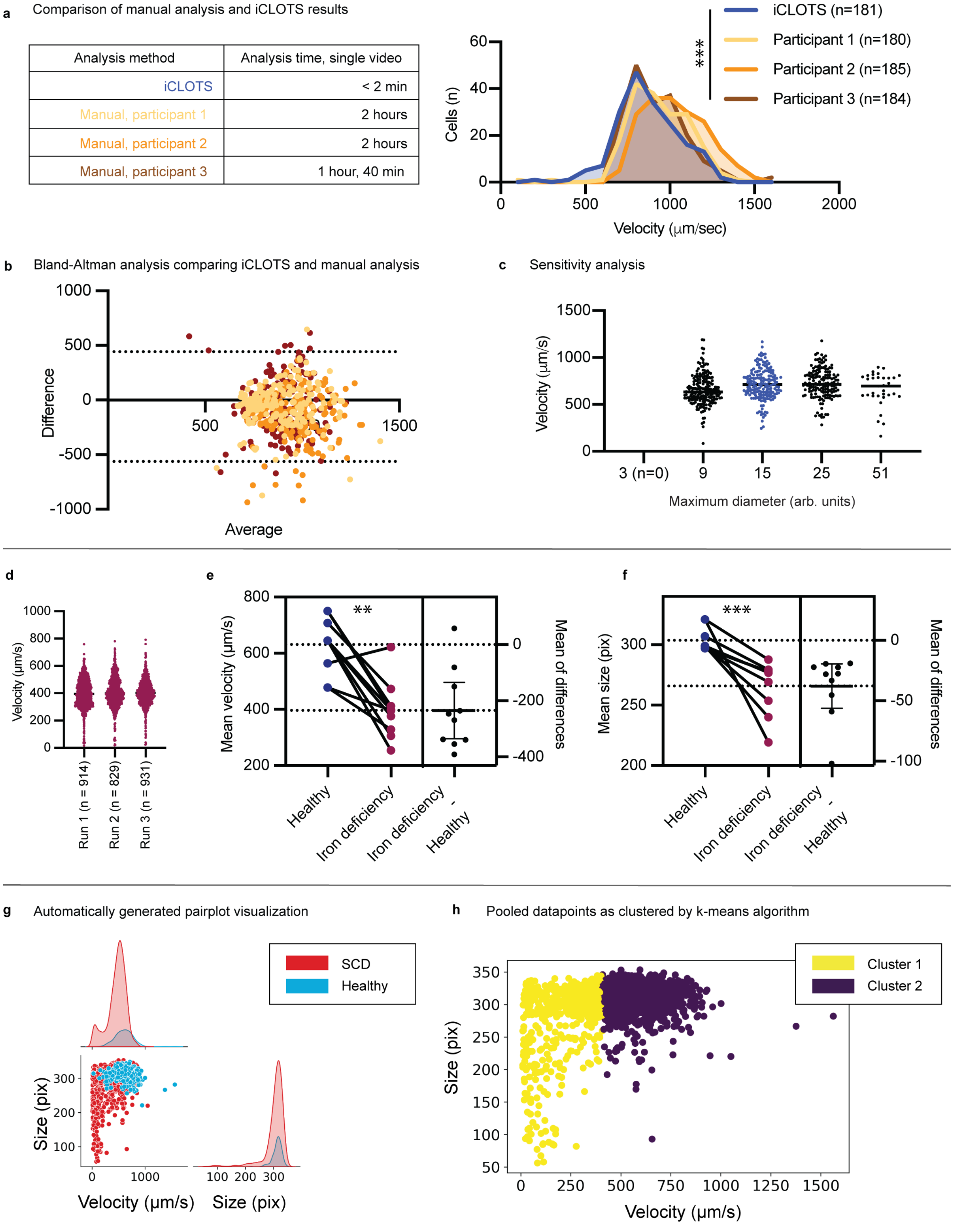

Supplementary figure 3. **iCLOTS single cell tracking application is robust, reduces analysis time, and produces data sufficiently detailed for machine learning analysis. a** iCLOTS generates single cell velocity values for individual cells in a fraction of the time required for manual analysis. While iCLOTS performed similarly to manual analysis, we find inter-participant variability in manual analysis as well, indicating potential for error in analyzing large datasets by hand (difference ***p<0.0001 by one-way ANOVA). **b** Bland-Altman plot analyzes the agreement between iCLOTS and each participant. **c** Sensitivity analysis shows analysis of the sample video performed with similar minimum cell diameter parameter values produces similar results. Parameter values chosen intentionally to exclude all data points result in no detected cells. Parameter values chosen intentionally to include image noise results in a reduced number of cells due to quality restrictions imposed on data points. **d** Single cell velocity measurements from three separate experimental trials with RBCs from the same iron deficiency anemia sample are not significantly different (n = 914, 829, and 931 RBCs, respectively, p=0.24 by ANOVA). **e** An estimation plot, a method of presenting a t-test that displays raw data and the confidence interval for the difference between means, shows iron deficiency anemia RBC samples have a significantly lower mean cell velocity than their time-matched healthy control RBC samples (n=10 pairs, **p=0.0005 via two-sided paired t test, data presented as mean values +/- standard deviation). **f** Iron deficiency anemia RBC samples also have a significant lower mean cell size than their time-matched healthy control RBC samples (n=10 pairs, ***p=0.0012 via two-sided paired t test, data presented as mean values +/- standard deviation). **g** Automatically generated pairplots output with final data allow software users to quickly parse differences between analyzed conditions. **h** When all data points from all conditions are combined, k-means algorithms with a software-user input $n_{clusters}$ cluster these data points into groups describing numerically similar data points. Here RBCs are grouped into low- and high-velocity clusters based on optimized k-means calculations. Source data are provided as a Source Data file.

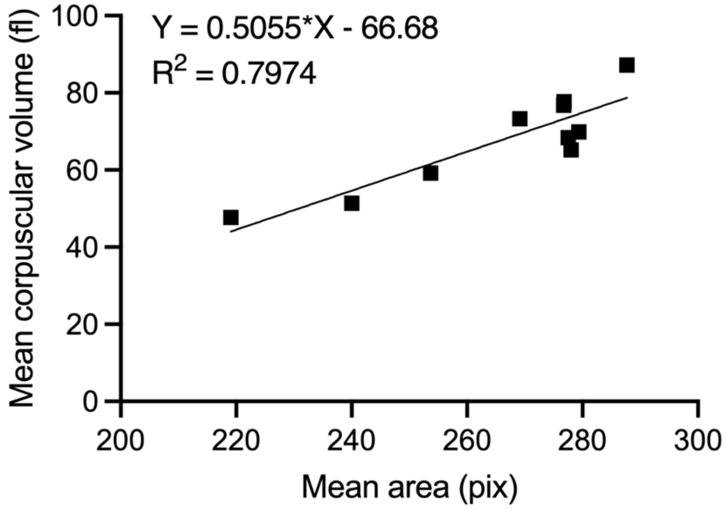

Supplementary figure 4. **Metrics calculated by iCLOTS correspond with clinical gold-standard measurements.** Mean RBC area (pixels) for nine patient blood samples meeting the criteria for iron deficiency anemia were compared to mean corpuscular volume (MCV) measurements taken by complete blood count (CBC). An increase in cell area measurements corresponds with an increase in MCV. Source data are provided as a Source Data file.

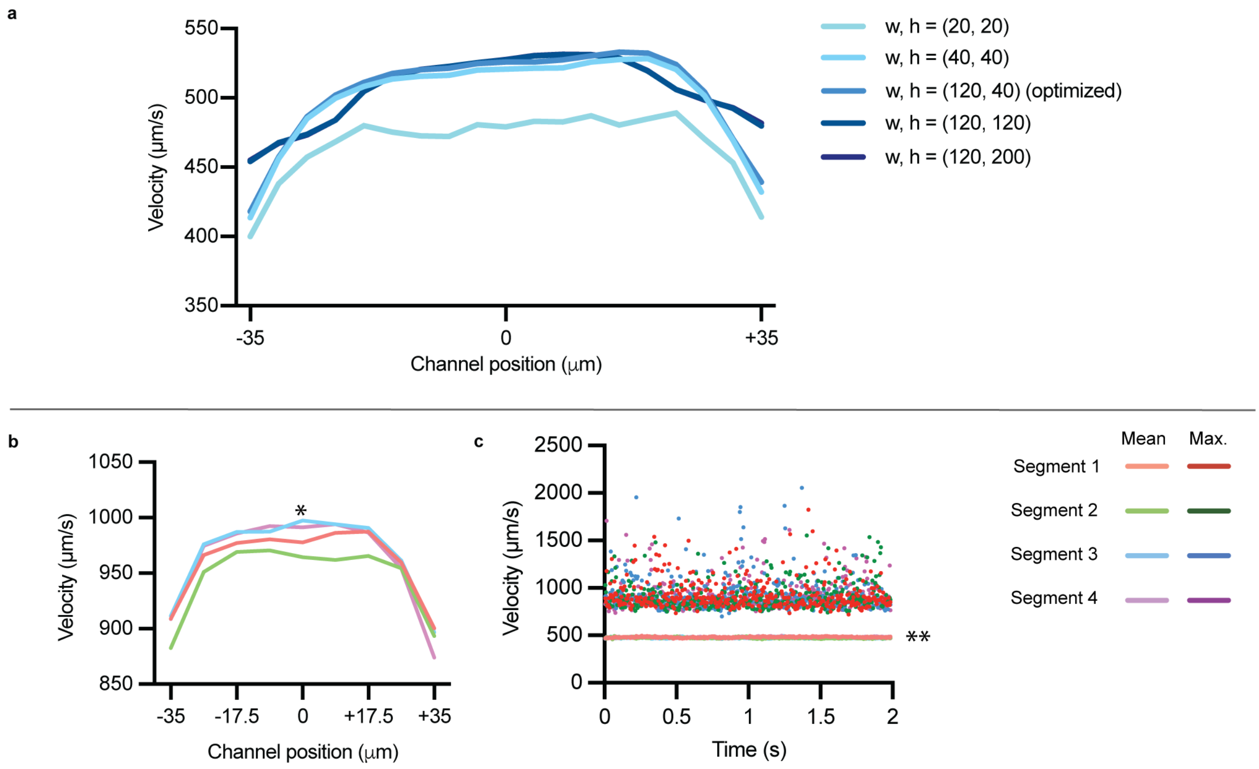

Supplementary figure 5. **iCLOTS velocity profile quantification application is robust in microfluidic devices including a novel microfluidic platform for quantification of oxygen-dependent viscosity. a** Shi-Tomasi corner detection finds patterns pixel intensity typically representing a cluster of cells within the channel. Kanade-Lucas-Tomasi optical flow algorithms connect these patterns of cells across frames into individual trajectories. Window size is a crucial user-input algorithm parameter describing the pixel distance in x, y directions that a detected pattern is searched for in the subsequent frame. Sensitivity analysis shows that a range of window sizes can accurately detect velocity profiles, but small windows (here, [20, 20]) may miss fastest-moving patterns, resulting in an erroneous profile blunting. Reasonable window size values ([40, 40], [120, 40], [120, 120], and [200, 200]) do not produce significantly different profiles (p=0.90 by ANOVA). Repeatability analysis performed on **b** profiles and **c** time course data for four sequential segments of a single 8 second video show potential for variability in results from microfluidic devices over time. Mean velocity profiles (n=4 segments, *p=0.0021 via paired one-way ANOVA with Geisser-Greenhouse correction) and mean velocity time course series (n=4 segments, **p<0.0001 via one-way ANOVA) were significantly different. Maximum velocity values per frame were not significantly different (n=4 segments, p=0.48). Source data are provided as a Source Data file.

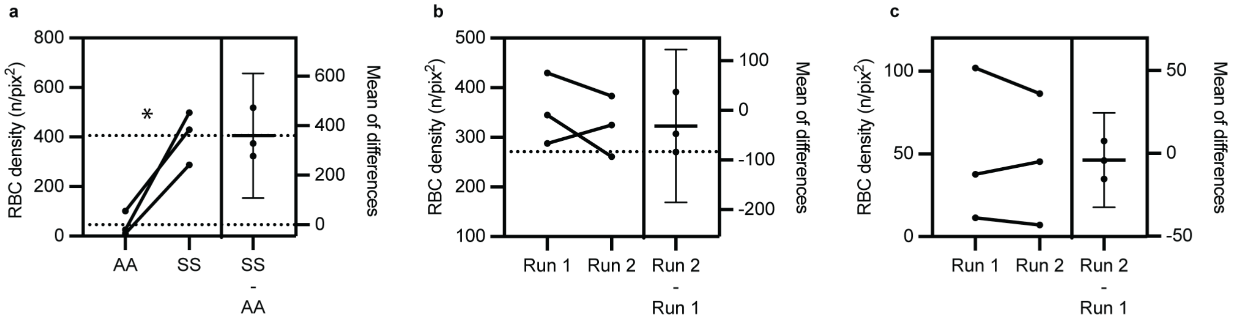

Supplementary figure 6. **Repeatable brightfield microscopy analysis shows healthy control RBCs are less adhesive than SCD RBCs on laminin surfaces. a** An estimation plot shows healthy control (AA) RBC samples adhere to laminin-coated microfluidic channels to a lesser degree than time-matched SCD (SS) RBC samples (n=3 paired samples, *p=0.026 by two-sided paired t test). Additional estimation plots show repeated runs of the same RBC sample for both **b** healthy controls (n=3 paired experiments) and **c** SCD samples (n=3 paired experiments) are not significantly different (p=0.60 and p=0.47, respectively). Data are presented as mean values +/- standard deviation. Source data are provided as a Source Data file.

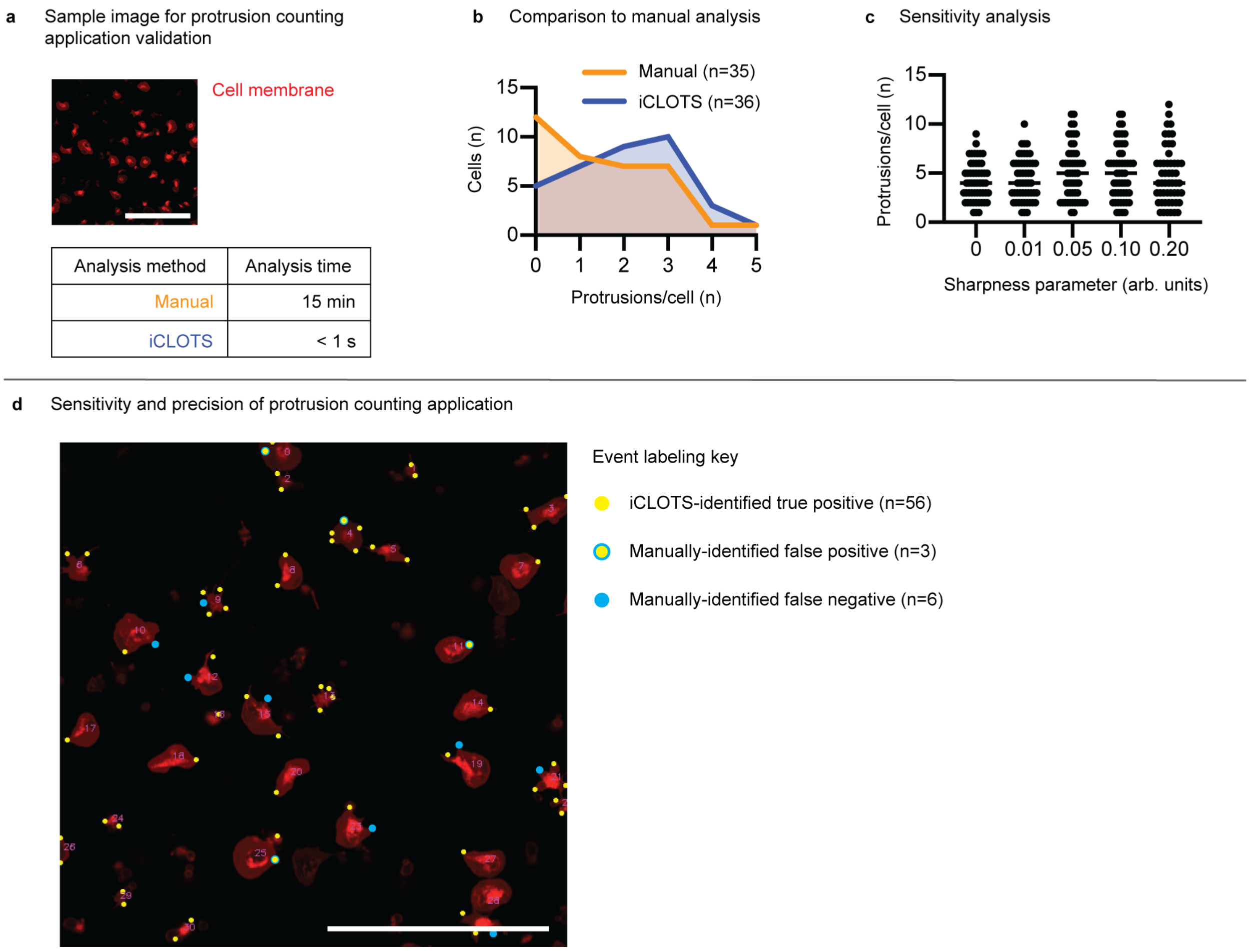

Supplementary figure 7. **Specialized iCLOTS protrusion counting application generates a single-cell resolution filopodia-like protrusion count for individual cells. a** Number of protrusions per cell is quantified in an image of fluorescently stained platelets in a fraction of the time required for manual analysis (n=1 experiment). Data taken at 100x magnification, scale bars represent 50 μm. **b** In this instance, iCLOTS analysis tends to overestimate number of filopodia-like protrusions as compared to manual analysis. **c** Sensitivity analysis shows analysis of the sample image performed with similar corner sharpness, or how distinct a protrusion must be from the main body of the cell, parameter values does not produce statistically significant changes in results (p=0.22 by ANOVA). Higher values of sharpness, indicating more permissiveness, result in higher numbers of filopodia-like protrusions detected. **d** Manual protrusion characterization can be subjective. iCLOTS applies uniform criteria across an image, increasing reliability and repeatability of results. Here, as compared to a manual analysis performed by a trained hematologist, iCLOTS had a sensitivity of 95% and a precision of 90% (n=1 experiment). Data taken at 100x magnification, scale bars represent 100 μm. Source data are provided as a Source Data file.

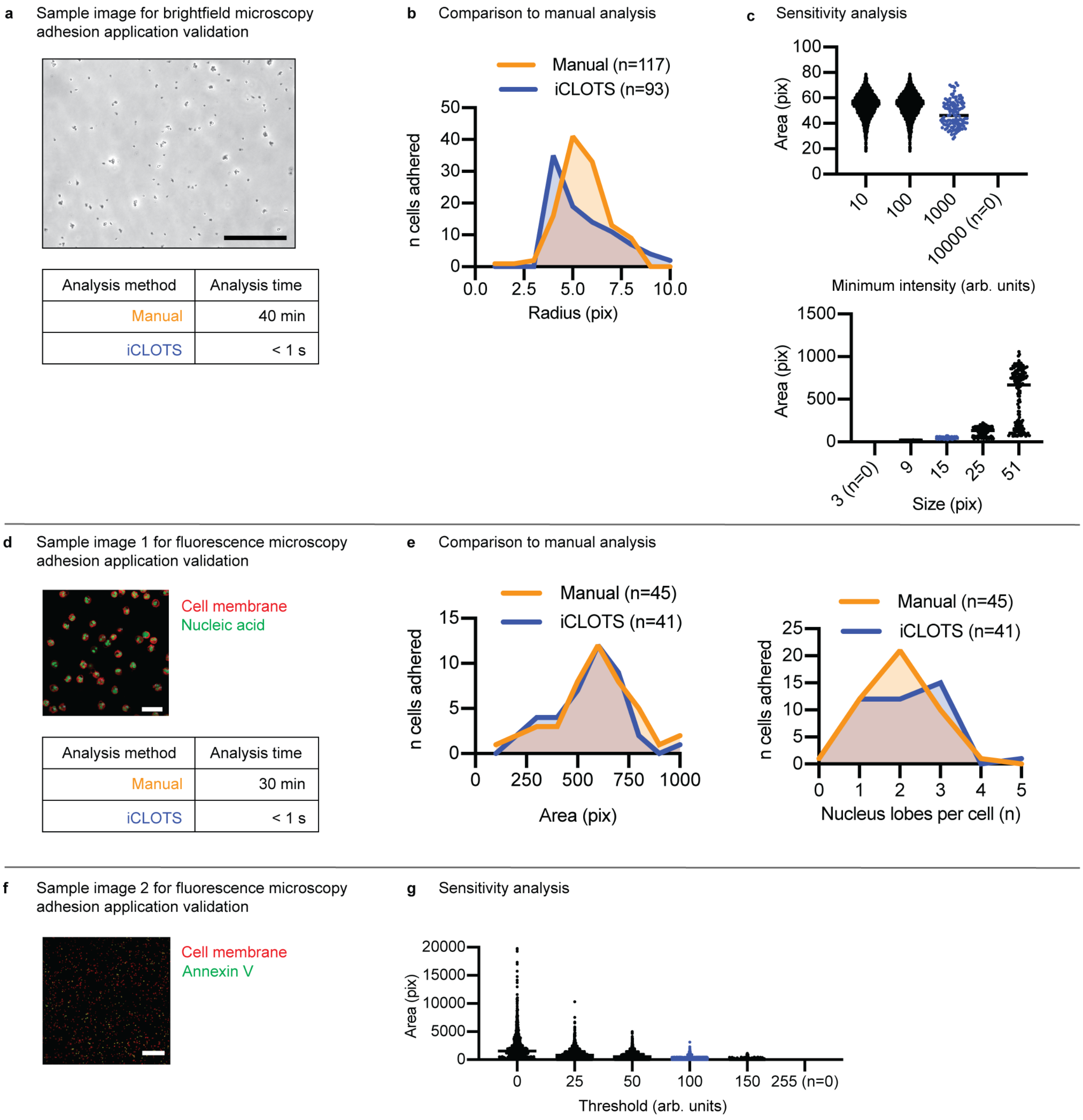

Analysis method	Analysis time
Manual	40 min
iCLOTS	< 1 s

Analysis method	Analysis time
Manual	30 min
iCLOTS	< 1 s

Supplementary figure 8. **iCLOTS suite of adhesion applications are robust and reduce analysis time. a** iCLOTS brightfield microscopy adhesion application generates area and circularity measurements for individual cells, here in a sample image of platelets in a fraction of the time required for manual analysis (n=1 experiment). Data taken at 30x magnification, scale bars represent 100 µm. **b** Results from iCLOTS analysis were not statistically different from manually calculated results (p=0.57 by Mann-Whitney). **c** Sensitivity analysis shows analysis of the sample image performed with similar minimum cell pixel intensity parameter values produces similar results. Parameter values chosen intentionally to exclude all reasonable data points results in no results, showing software fidelity. **d** iCLOTS fluorescence microscopy adhesion application generates detailed

descriptors of cells including area, circularity, fluorescence intensity, and regions of a secondary stain, here in a sample image of fluorescently stained neutrophils in a fraction of the time required for manual analysis (n=1 experiment). Data taken at 40x magnification, scale bars represent 25 μm. **e** Cell area measurements and nucleus lobe count from a sample image from iCLOTS analysis were not statistically different from manual tests (p=0.82 and p=0.15 by Mann Whitney, respectively). **f** A sample image to be used for sensitivity analysis shows images of platelets is stained for cell membrane (n=1 experiment). Data taken at 40x magnification, scale bars represent 200 μm. **g** Sensitivity analysis shows analysis of the sample image performed with a reasonable range of threshold parameter values produces similar results. Parameter values chosen intentionally to exclude all data points result in no detected cells. Parameter values chosen intentionally to include image noise results in many large, spurious data points being detected. Source data are provided as a Source Data file.

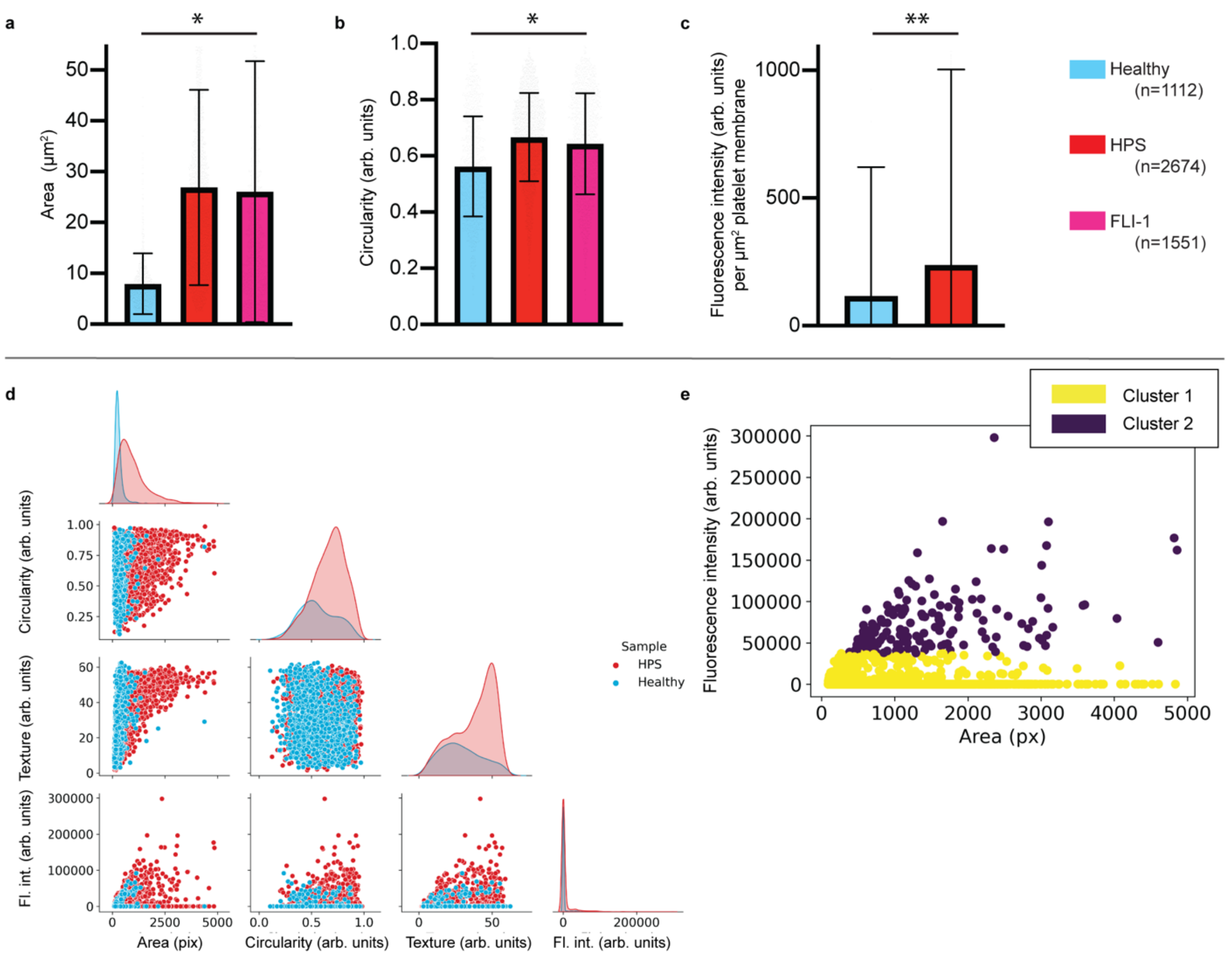

Supplementary figure 9. **Single-cell resolution analysis of cells adhered to collagen-coated surfaces show differences between healthy control and samples from platelets with blood disorders. a** HPS platelets (n=2,674 platelets) and FLI-1 platelets (n=1,551), are significantly larger than healthy control platelets (n=1,112 platelets), indicating increased platelet spreading (*p<0.0001 by Kruskal-Wallis). **b** HPS and FLI-1 platelets from the same experiment are significantly more round than healthy control platelets (*p<0.0001 by Kruskal-Wallis). **c** HPS platelets from the same experiment show more phosphatidylserine exposure as indicated by fluorescence intensity of annexin V staining (**p<0.0001 by two-sided Mann-Whitney). All data are presented as mean values +/- standard deviation. **d** iCLOTS automatically generates pairplots of multi-factorial healthy control and HPS data sets to allow software users to quickly parse differences between analyzed conditions. **e** When all data points from all conditions are combined, k-means algorithms with a software-user input $n_{clusters}$ cluster these data points into groups describing numerically similar data points. Here, grouped platelets are automatically divided into low and high levels of phosphatidylserine exposure based on optimized k-means calculations. Source Data are provided as a Source Data file.

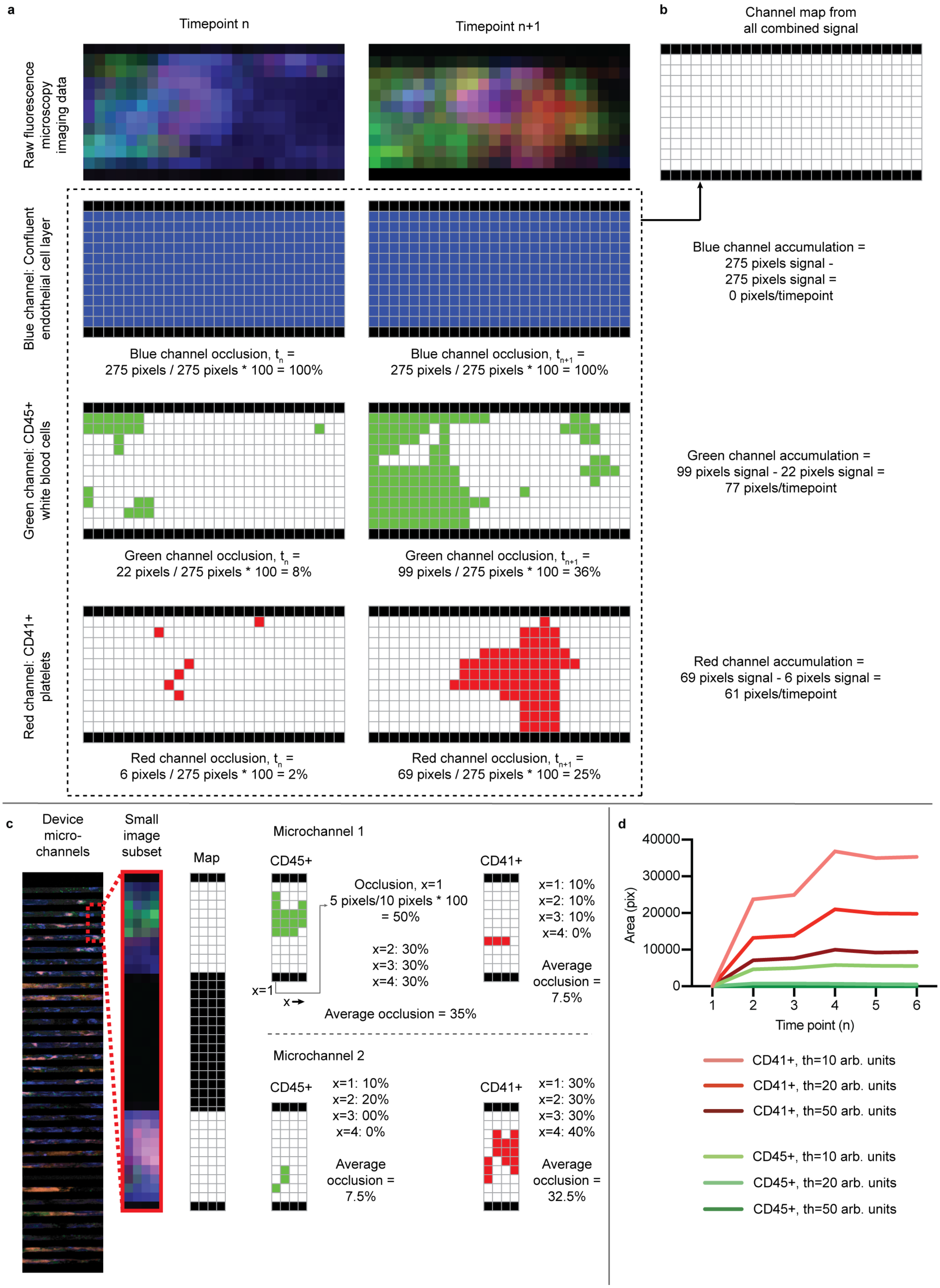
a
Timepoint n
Timepoint n+1
b
Channel map from all combined signal
Raw fluorescence microscopy imaging data
Blue channel: Confluent endothelial cell layer
Blue channel occlusion, t_n = 275 pixels / 275 pixels * 100 = 100%
Blue channel occlusion, t_{n+1} = 275 pixels / 275 pixels * 100 = 100%
Blue channel accumulation = 275 pixels signal - 275 pixels signal = 0 pixels/timepoint
Green channel: CD45+ white blood cells
Green channel occlusion, t_n = 22 pixels / 275 pixels * 100 = 8%
Green channel occlusion, t_{n+1} = 99 pixels / 275 pixels * 100 = 36%
Green channel accumulation = 99 pixels signal - 22 pixels signal = 77 pixels/timepoint
Red channel: CD41+ platelets
Red channel occlusion, t_n = 6 pixels / 275 pixels * 100 = 2%
Red channel occlusion, t_{n+1} = 69 pixels / 275 pixels * 100 = 25%
Red channel accumulation = 69 pixels signal - 6 pixels signal = 61 pixels/timepoint
c
Device micro-channels
Small image subset
Map
Microchannel 1
CD45+
Occlusion, x=1
5 pixels/10 pixels * 100 = 50%
x=2: 30%
x=3: 30%
x=4: 30%
x=1
x
Average occlusion = 35%
CD41+
x=1: 10%
x=2: 10%
x=3: 10%
x=4: 0%
Average occlusion = 7.5%
Microchannel 2
CD45+
x=1: 10%
x=2: 20%
x=3: 00%
x=4: 0%
Average occlusion = 7.5%
CD41+
x=1: 30%
x=2: 30%
x=3: 30%
x=4: 40%
Average occlusion = 32.5%
d
Area (pix)
40000
30000
20000
10000
0
1
2
3
4
5
6
Time point (n)
CD41+, th=10 arb. units
CD41+, th=20 arb. units
CD41+, th=50 arb. units
CD45+, th=10 arb. units
CD45+, th=20 arb. units
CD45+, th=50 arb. units

Supplementary figure 10. **iCLOTS multiscale microfluidic accumulation applications rely on pixel thresholds and indexing to quantify patterns of cell and particle deposition on potentially complex surfaces. a** As time progresses, CD45+ white blood cells (green) and CD41+ platelets (red) from a clinical whole blood sample from a patient with SCD accumulate on a cultured endothelial monolayer (Cell mask, blue) within a microfluidic device. A user-defined threshold for each present color channel converts image layers of varying pixel intensity into a binary image. **b** Signal from red, green, and blue color channels at all timepoints is combined to create a channel map. The number of pixels within the map (here, 275 pixels) is set as the channel area. iCLOTS provides an option to input a micron-to-pixel ratio to convert final pixel area-based measurements to μm^2. While this figure uses a simple channel as an example, iCLOTS can create a map for any number of complicated geometries. Staining the endothelial cell monolayer provides a clear background for the channel, improving map accuracy. Signal area is divided by channel area to create a percent occlusion for the region of interest. Accumulation between subsequent frames is calculated as the change in signal area from one frame to the next. These calculations are completed for each selected color channel. **c** A specialized microchannel application quantifies a spatial percent occlusion for one or many straight-channel microchannels. A map is created, thresholds are applied, and occlusion is calculated as y-area of signal divided by channel height at each x position within the channel for each color. Descriptive statistics including average occlusion for a channel at each time point are provided. **d** As less signal is quantified as threshold increases, user-set threshold does have an effect on final accumulation and occlusion values. Sensitivity analysis of full-microfluidic device timecourse data shows that while higher threshold values (th) detect less fluorescence microscopy stain signal, a pattern of increasing occlusion within the microfluidic device over time remains. Source data are provided as a Source Data file.

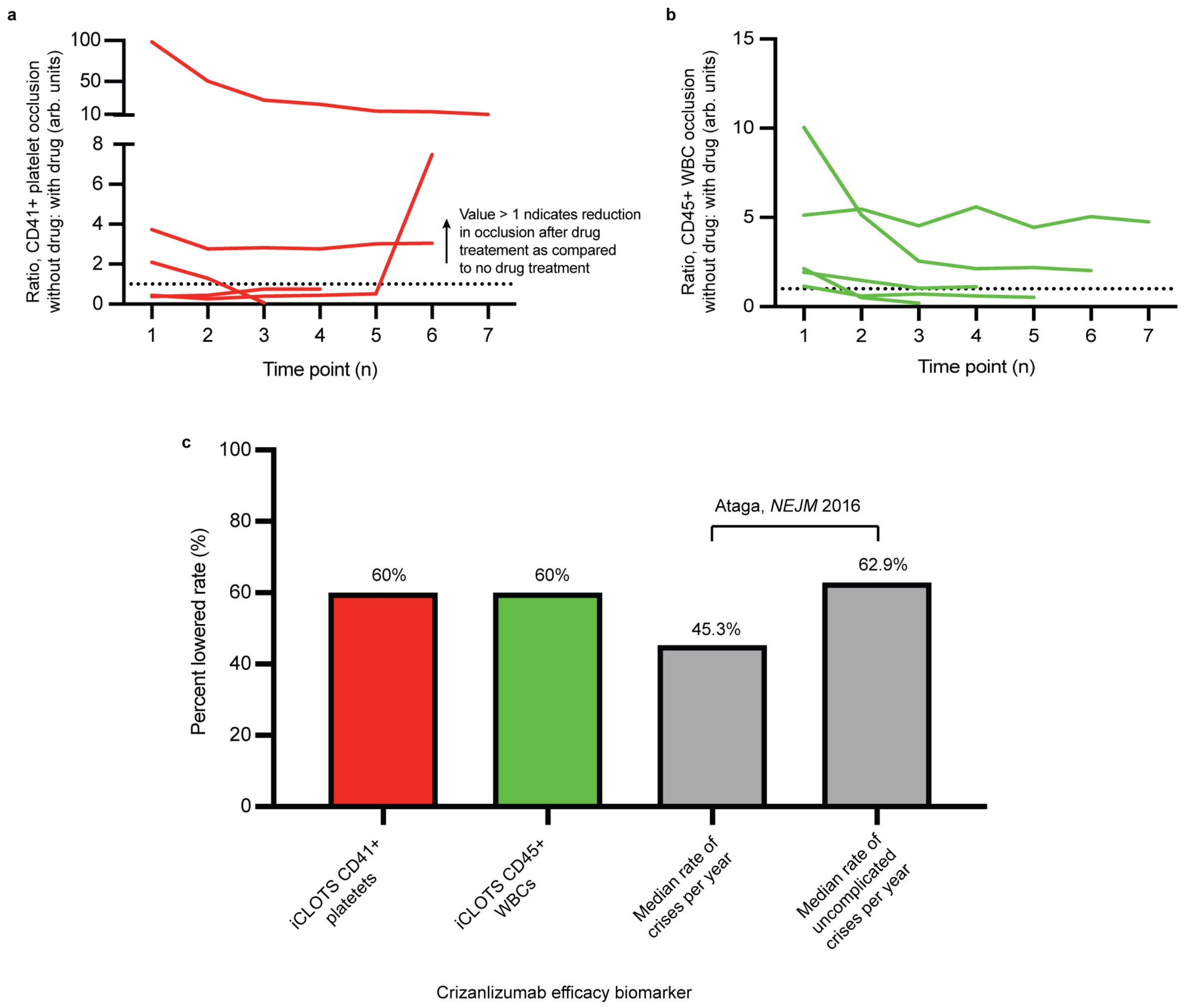

Supplementary figure 11. **iCLOTS analyses results serve as effective biomarkers of pathophysiology congruent with findings from successful clinical trials.** In experiments investigating the accumulation of CD41+ platelets and CD45+ white blood cells in a microvasculature-on-a-chip model (n=5 experiments), a ratio of microchannel occlusion without crizanlizumab treatment to microchannel occlusion with crizanlizumab treatment greater than 1 indicates that crizanlizumab is reducing the rate of microchannel occlusion within the microfluidic devices. Using the iCLOTS microfluidic device-scale accumulation and occlusion application, we find that in n=5 sickle cell disease patient samples, the ratio of **a** CD41+ platelet occlusion and **b** CD45+ white blood cell occlusion for treatment without crizanlizumab to treatment with crizanlizumab indicates reduction in occlusion (ratio > 1) in 60% of samples. **c** Ataga et al. (New England Journal of Medicine, 2016) found a similar lowered percentage rate of crises per year (1.63 with high-dose crizanlizumab treatment as compared with. 2.98 with placebo, indicating a statistically significant 45.3% reduction) and uncomplicated crises per year (1.08 with high-dose crizanlizumab treatment as compared with 2.91 with placebo, indicating a statistically significant 62.9% reduction.) Source data are provided as a Source Data file.

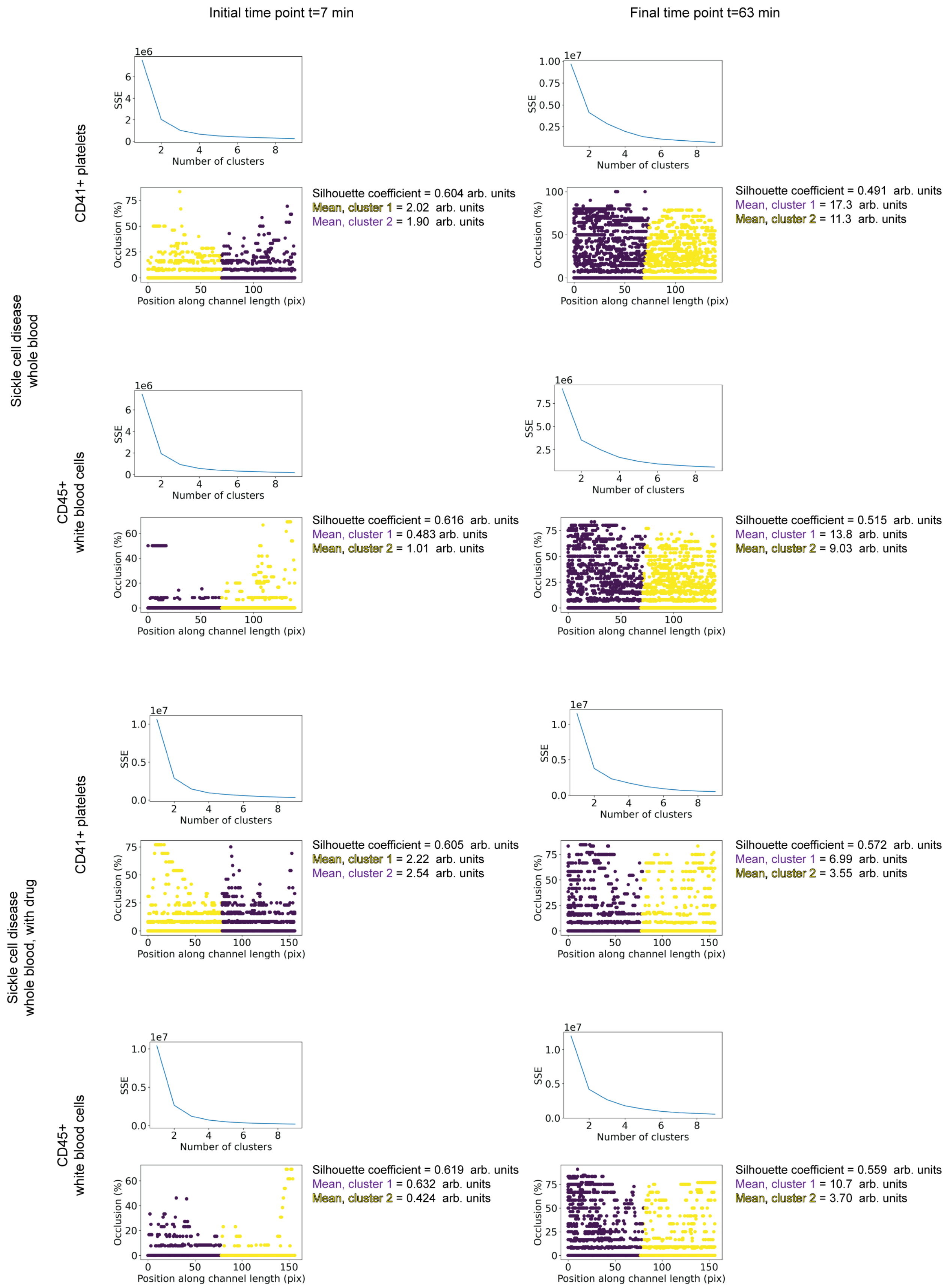
Initial time point t=7 min
Final time point t=63 min
Sickle cell disease whole blood
CD41+ platelets
Silhouette coefficient = 0.604 arb. units
Mean, cluster 1 = 2.02 arb. units
Mean, cluster 2 = 1.90 arb. units
Silhouette coefficient = 0.491 arb. units
Mean, cluster 1 = 17.3 arb. units
Mean, cluster 2 = 11.3 arb. units
CD45+ white blood cells
Silhouette coefficient = 0.616 arb. units
Mean, cluster 1 = 0.483 arb. units
Mean, cluster 2 = 1.01 arb. units
Silhouette coefficient = 0.515 arb. units
Mean, cluster 1 = 13.8 arb. units
Mean, cluster 2 = 9.03 arb. units
Sickle cell disease whole blood, with drug
CD41+ platelets
Silhouette coefficient = 0.605 arb. units
Mean, cluster 1 = 2.22 arb. units
Mean, cluster 2 = 2.54 arb. units
Silhouette coefficient = 0.572 arb. units
Mean, cluster 1 = 6.99 arb. units
Mean, cluster 2 = 3.55 arb. units
CD45+ white blood cells
Silhouette coefficient = 0.619 arb. units
Mean, cluster 1 = 0.632 arb. units
Mean, cluster 2 = 0.424 arb. units
Silhouette coefficient = 0.559 arb. units
Mean, cluster 1 = 10.7 arb. units
Mean, cluster 2 = 3.70 arb. units
SSE
Number of clusters
Occlusion (%)
Position along channel length (pix)

Supplementary figure 12. **K-means machine learning interpretation of occlusion patterns in microchannels for SCD whole blood and SCD whole blood samples treated with drug crizanlizumab show variable patterns of CD45+ WBC and CD41+ platelet accumulation over time.** Scree plot analysis "elbow" point quantitatively determines the best number of clusters to use in a k-means algorithm (top graph within sets). For all datasets from a single experiment comprised of data points described by the features percent occlusion and channel position, scree plot analysis indicates that these datasets should be divided into two clusters to best balance minimal clusters with minimum variance within clusters. Percent occlusion/channel position data points for each condition (with and without drug; at an initial and final timepoint; and CD41+ or CD45+ signal) are plotted (bottom graph within sets). K-means clustering with $n_{clusters}$=2, as prescribed by scree plot analysis, is performed. In all cases, the algorithm mathematically divides data points into beginning- and end-of-channel (proximal- and distal-, respectively) clusters based on calculations for optimized k-means clustering. The mean of each cluster and silhouette coefficient between clusters is provided. Silhouette coefficient is a metric used to calculate the goodness of a clustering technique that ranges from -1 to 1, with 1 indicating best cluster separation. Here, higher silhouette coefficient values indicate a greater difference between beginning- and end-of-channel clusters. In untreated SCD whole blood, CD45+ white blood cells accumulate preferentially at the end of channels at early timepoints, but this affect decreases over time. CD41+ platelets preferentially occlude at the beginning of channels in later time points. These patterns of occlusion are changed in SCD whole blood treated with P-selectin inhibitor Crizanlizumab. Source data are provided as a Source Data file.

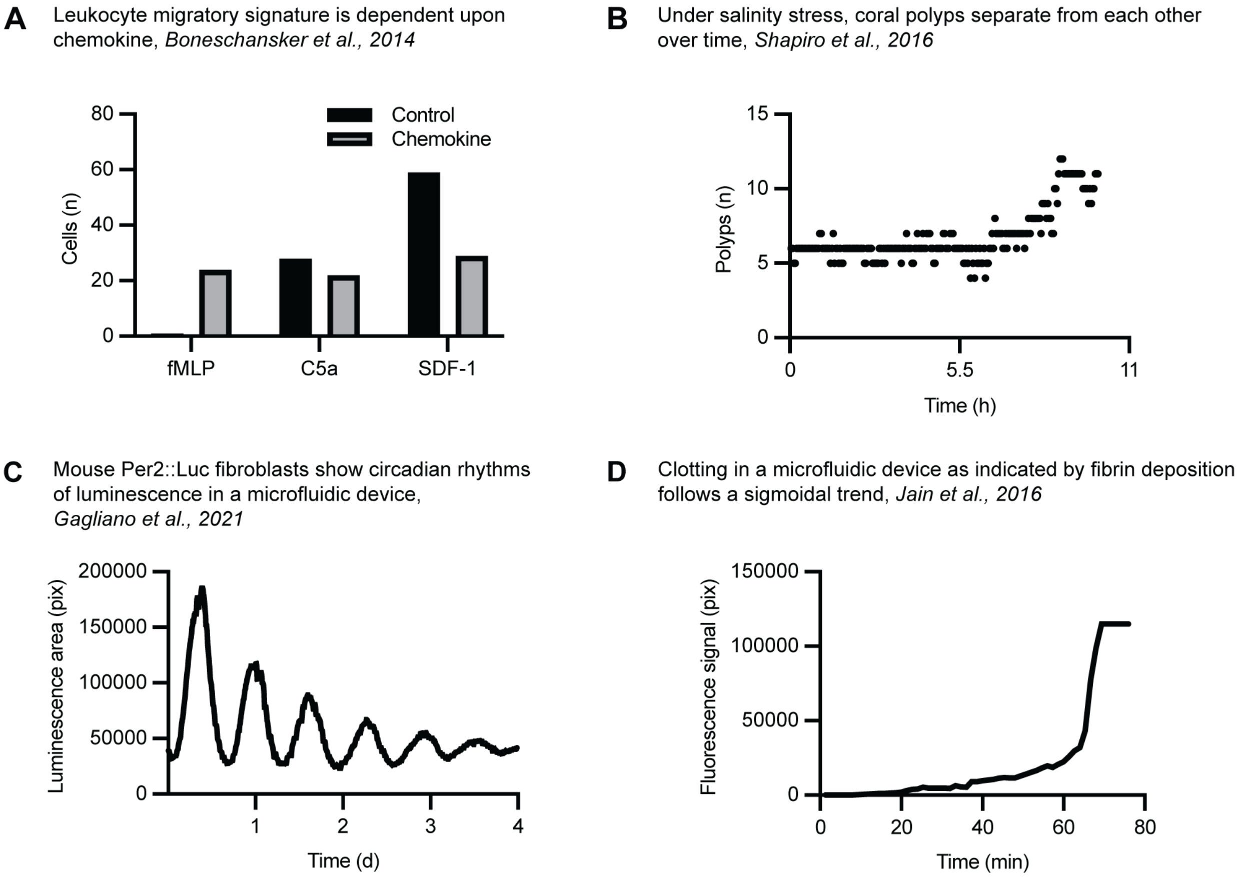

Supplementary figure 13. **Data generated from iCLOTS recapitulates key findings from previously published *Nature Communications* manuscripts. a** iCLOTS single cell tracking application counts cells in motion as they move away from (control solution) or towards (chemokine solution) a chemokine. As published in Boneschansker et al., 2014, we also find leukocytes in suspension migrate preferentially towards chemoattractant fMet-Leu-Phe (fMLP), that complement component 5a (C5a) induces both chemoattraction and repulsion in equal proportions in leukocytes, and that stromal cell-derived factor 1 (SDF-1) primarily repulses lymphocytes. **b** iCLOTS brightfield microscopy adhesion application counts coral polyps over time within a coral-on-a-chip microfluidic system, also indicating potential for use with other cells and/or multicellular structures. As published in Shapiro et al., 2016, we also find a gradual increase in salinity applied over time causes coral polyps to separate from each other. **c** iCLOTS accumulation and occlusion application quantifies fluorescence microscopy signal from a microfluidic device designed to recreate the circadian system. As published in Gagliano et al., 2021, we also find that by controlling the timing, period, and frequency of metabolic perturbations, circadian gene expression as detected by luminescence oscillates over time. **d** iCLOTS accumulation and occlusion application quantifies fibrin deposition in a microfluidic device. As published in Jain et al., 2016, we also find that fibrin deposition from whole blood onto a microfluidic surface follows a sigmoidal trend. Source data are provided as a Source Data file.

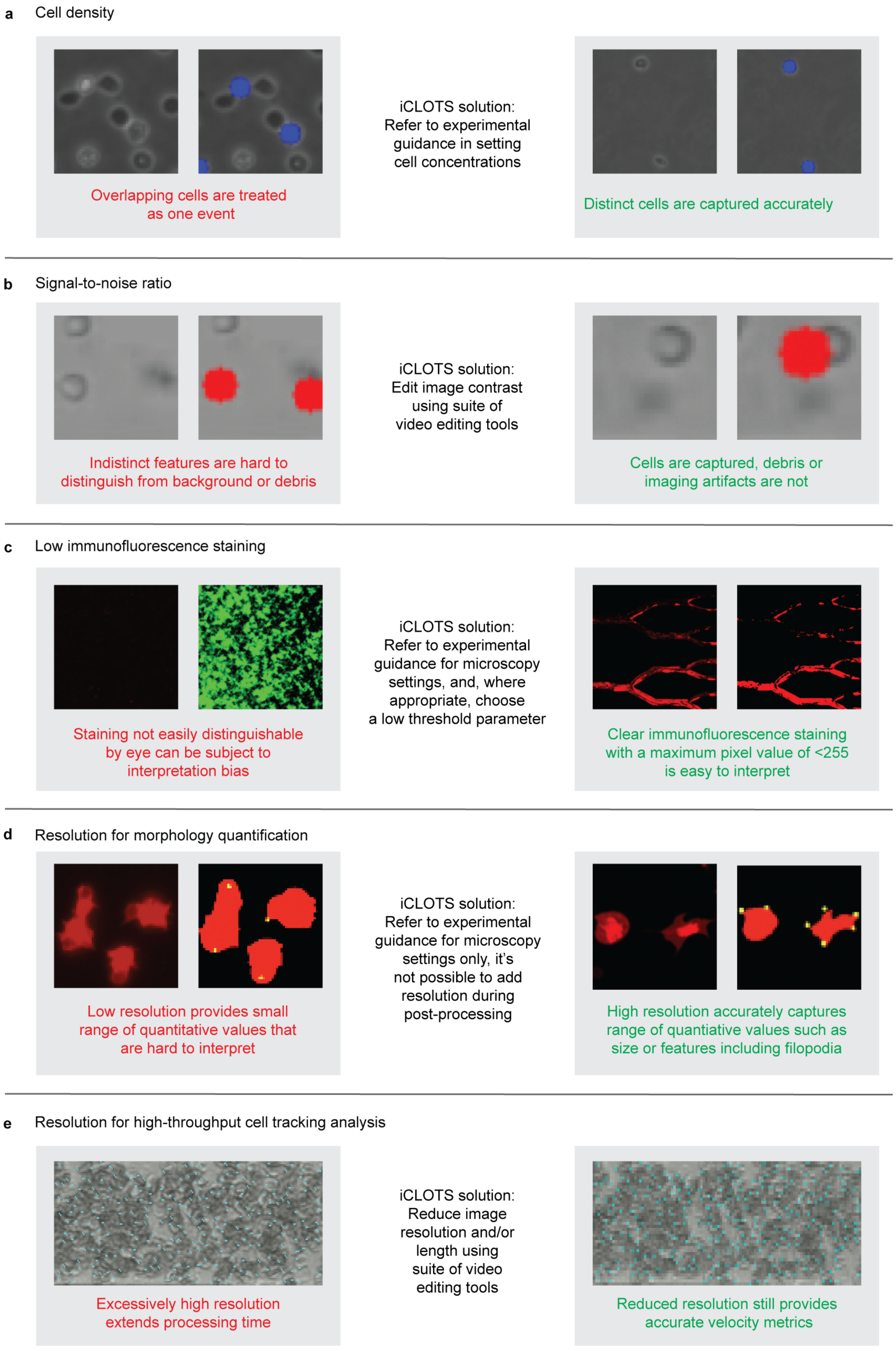
a
Cell density
Overlapping cells are treated as one event
iCLOTS solution: Refer to experimental guidance in setting cell concentrations
Distinct cells are captured accurately
b
Signal-to-noise ratio
Indistinct features are hard to distinguish from background or debris
iCLOTS solution: Edit image contrast using suite of video editing tools
Cells are captured, debris or imaging artifacts are not
c
Low immunofluorescence staining
Staining not easily distinguishable by eye can be subject to interpretation bias
iCLOTS solution: Refer to experimental guidance for microscopy settings, and, where appropriate, choose a low threshold parameter
Clear immunofluorescence staining with a maximum pixel value of <255 is easy to interpret
d
Resolution for morphology quantification
Low resolution provides small range of quantitative values that are hard to interpret
iCLOTS solution: Refer to experimental guidance for microscopy settings only, it's not possible to add resolution during post-processing
High resolution accurately captures range of quantiative values such as size or features including filopodia
e
Resolution for high-throughput cell tracking analysis
Excessively high resolution extends processing time
iCLOTS solution: Reduce image resolution and/or length using suite of video editing tools
Reduced resolution still provides accurate velocity metrics

Supplementary figure 14. **Common data quality issues can be solved using iCLOTS experimental guidance or the iCLOTS suite of video editing tools in order to improve final feature quantification.** The most common data quality issues the iCLOTS development team encounters are: **a** too high a cell density, which may be solved by reducing overall cell concentration; **b** a low signal-to-noise ratio, which may be solved by adjusting imaging data contrast values; **c** low signal from immunofluorescence staining, which may be solved by calibrating staining procedures and microscopy settings; **d** too low a resolution for quantifying morphological features, which may be solved by choosing a higher-magnification microscope objective; or, **e** too high a resolution for high-throughput data analysis, which may be solved by reducing file resolution and/or length.

Supplementary references

1 Rosenbluth, M. J., Lam, W. A. & Fletcher, D. A. Analyzing cell mechanics in hematologic diseases with microfluidic biophysical flow cytometry. *Lab Chip* **8**, 1062-1070, doi:10.1039/b802931h (2008).
2 Banton, S. *Human peripheral reticulocte isolation and exosome release in vitro* Doctor of Philosophy thesis, Georgia Institute of Technology, Emory University, (2017).
3 Singer, M. *et al.* The Third International Consensus Definitions for Sepsis and Septic Shock (Sepsis-3). *JAMA* **315**, 801-810, doi:10.1001/jama.2016.0287 (2016).
4 Bennett, M. *et al.* Molecular clutch drives cell response to surface viscosity. *Proceedings of the National Academy of Sciences* **115**, 1192-1197, doi:10.1073/pnas.1710653115 (2018).
5 Myers, D. R. *et al.* Endothelialized microfluidics for studying microvascular interactions in hematologic diseases. *J Vis Exp*, doi:10.3791/3958 (2012).
6 McKinney, W. *Data Structures for Statistical Computing in Python*. (2010).
7 Hunter, J. D. Matplotlib: A 2D Graphics Environment. *Computing in Science & Engineering* **9**, 90-95, doi:10.1109/MCSE.2007.55 (2007).
8 Waskom, M. L. seaborn: statistical data visualization. *Journal of Open Source Software* **6**, 3021, doi:10.21105/joss.03021 (2021).
9 Pedregosa, F. *et al.* Scikit-learn: Machine Learning in Python. *J. Mach. Learn. Res.* **12**, 2825–2830 (2011).
10 Lloyd, S. Least squares quantization in PCM. *IEEE Transactions on Information Theory* **28**, 129-137, doi:10.1109/TIT.1982.1056489 (1982).
11 Makles, A. Stata tip 110: How to get the optimal k-means cluster solution. *The Stata Journal* **12**, 347-351 (2012).
12 Rousseeuw, P. J. Silhouettes: A graphical aid to the interpretation and validation of cluster analysis. *Journal of Computational and Applied Mathematics* **20**, 53-65, doi:https://doi.org/10.1016/0377-0427(87)90125-7 (1987).
