# Supplementary material for: iCLOTS: open-source, artificial intelligence-enabled software for analyses of blood cells in microfluidic and microscopy-based assays
Source: Nat Commun. 2023 Aug 18;14:5022. doi: 10.1038/s41467-023-40522-4 (PMC10439163; doi:10.1038/s41467-023-40522-4)
Supplement: Supplementary file 3 — Reporting Summary [file 41467_2023_40522_MOESM3_ESM.pdf]

nature portfolio

Corresponding author(s): Wilbur Lam, MD, PhD

Last updated by author(s): Feb 21, 2023

Reporting Summary

Nature Portfolio wishes to improve the reproducibility of the work that we publish. This form provides structure for consistency and transparency in reporting. For further information on Nature Portfolio policies, see our Editorial Policies and the Editorial Policy Checklist.

Statistics

For all statistical analyses, confirm that the following items are present in the figure legend, table legend, main text, or Methods section.

n/a	Confirmed	
☐	☒	The exact sample size (n) for each experimental group/condition, given as a discrete number and unit of measurement
☐	☒	A statement on whether measurements were taken from distinct samples or whether the same sample was measured repeatedly
☐	☒	The statistical test(s) used AND whether they are one- or two-sided<br>*Only common tests should be described solely by name; describe more complex techniques in the Methods section.*
☐	☒	A description of all covariates tested
☐	☒	A description of any assumptions or corrections, such as tests of normality and adjustment for multiple comparisons
☐	☒	A full description of the statistical parameters including central tendency (e.g. means) or other basic estimates (e.g. regression coefficient) AND variation (e.g. standard deviation) or associated estimates of uncertainty (e.g. confidence intervals)
☐	☒	For null hypothesis testing, the test statistic (e.g. F, t, r) with confidence intervals, effect sizes, degrees of freedom and P value noted<br>*Give P values as exact values whenever suitable.*
☒	☐	For Bayesian analysis, information on the choice of priors and Markov chain Monte Carlo settings
☒	☐	For hierarchical and complex designs, identification of the appropriate level for tests and full reporting of outcomes
☒	☐	Estimates of effect sizes (e.g. Cohen's d, Pearson's r), indicating how they were calculated

Our web collection on statistics for biologists contains articles on many of the points above.

Software and code

Policy information about availability of computer code

Data collection	Microscopy acquisition software specific to the microscope used was used to collect imaging and video data.
Data analysis	This paper describes a piece of custom, standalone, open-source software titled "iCLOTS". iCLOTS methods are provided as scripts at github.com/LamLabEmory. iCLOTS source code is provided at github.com/iCLOTS. iCLOTS standalone software is provided for Mac and Windows operating systems at iCLOTS.org/software. iCLOTS methods are built upon many open-source Python libraries, which are described and cited in the manuscript and supplement. These libraries include: OpenCV version 4.5.3, Trackpy version 0.5.0, Scikit-image version 0.18.3, pandas version 1.3.3, Matplotlib version 3.4.3, Seaborn version 0.11.2, and Scikit-learn version 1.0.2. Information for accessing software and source code is included in the manuscript and supplement. Packages used for data analysis are described in the supplement. Manual analyses used to assess software accuracy were performed with measurement tools available within the image acquisition toolbox ofFIJI version 2.1.0.

For manuscripts utilizing custom algorithms or software that are central to the research but not yet described in published literature, software must be made available to editors and reviewers. We strongly encourage code deposition in a community repository (e.g. GitHub). See the Nature Portfolio guidelines for submitting code & software for further information.

Data

Policy information about availability of data

All manuscripts must include a data availability statement. This statement should provide the following information, where applicable:

- Accession codes, unique identifiers, or web links for publicly available datasets
- A description of any restrictions on data availability
- For clinical datasets or third party data, please ensure that the statement adheres to our policy

A subset of test data for every iCLOTS application is provided at iCLOTS.org/software and at github.com/LamLabEmory. All data analyzed in the manuscript is available without restrictions upon request to the corresponding author, Wilbur Lam, MD, PhD. (email: wilbur.lam@emory.edu)

Human research participants

Policy information about studies involving human research participants and Sex and Gender in Research.

Reporting on sex and gender	Information on sex and gender of control and clinical samples could be obtained by clinical collaborators if needed. Experimental results reported in the iCLOTS manuscript are presented primarily to demonstrate image analysis and machine learning software capabilities.
Population characteristics	All healthy control samples were collected from adult volunteers. All clinical samples were collected from pediatric patients with the diagnosis specified at the children's hospital specific to the assay performed, listed in the supplemental methods. Experimental results reported in the iCLOTS manuscript are presented primarily to demonstrate image analysis and machine learning software capabilities.
ecruitment	All healthy human blood control samples were collected from adult volunteers working within a research laboratory setting. These adult volunteers were primarily in their 20s and have no known health issues relevant to the studies performed. Most clinical samples were from pediatric patients, which may introduce bias. All clinical human blood samples were collected from patients at our affiliate hospitals specific to the assay performed, listed in the supplemental methods. Experimental results reported in the iCLOTS manuscript are presented primarily to demonstrate image analysis and machine learning software capabilities. No participants were compensated.
thics oversight	Study protocol was approved according to IRB from Georgia Institute of Technology, Children's Minnesota Hospital, University of Minnesota, and Emory University (see above). Specific IRB used for each assay is detailed in the supplemental data. Consent for all healthy human blood samples was obtained according to Georgia Institute of Technology IRB H15258.

Note that full information on the approval of the study protocol must also be provided in the manuscript.

Field-specific reporting

Please select the one below that is the best fit for your research. If you are not sure, read the appropriate sections before making your selection.

☒ Life sciences ☐ Behavioural & social sciences ☐ Ecological, evolutionary & environmental sciences

For a reference copy of the document with all sections, see nature.com/documents/nr-reporting-summary-flat.pdf

Life sciences study design

All studies must disclose on these points even when the disclosure is negative.

ample size	Experimental results reported in the iCLOTS manuscript are presented primarily to demonstrate image analysis and machine learning software capabilities, so no calculation of sample size was performed. Because findings were not presented as conclusive these sample sizes are sufficient.
Data exclusions	iCLOTS analyses, like all computational analyses, all rely on high-quality microscopy data. If microscopy quality data was poor (inconsistent illumination, excessive debris from experimental methods, clear defects in microfluidic channels), data was excluded. No data was excluded on the basis of results obtained only.
eplication	Experimental results were formed from multiple independent experiments, with all relevant n listed in experimental results. iCLOTS is a multi-lab collaboration. Software methods were verified by comparing computational results from multiple labs and by comparing computational results to manual analyses by expert researchers and clinicians. All attempts at replication were successful.
andomization	Experimental results reported in the iCLOTS manuscript designed primarily to demonstrate image analysis and machine learning software capabilities were performed on human blood samples obtained from healthy adult volunteers in an unbiased manner. All novel iCLOTS results were divided into healthy control and clinical patient sample groupings based on patient diagnosis made by clinicians.
Blinding	While the researchers who collected the example data had prior knowledge about the samples, the designers of the software were blinded at all points of data analysis.

Reporting for specific materials, systems and methods

We require information from authors about some types of materials, experimental systems and methods used in many studies. Here, indicate whether each material, system or method listed is relevant to your study. If you are not sure if a list item applies to your research, read the appropriate section before selecting a response.

Materials & experimental systems

n/a	Involved in the study	
☐	☒	Antibodies
☐	☒	Eukaryotic cell lines
☒	☐	Palaeontology and archaeology
☒	☐	Animals and other organisms
☒		Clinical data
☒	☐	Dual use research of concern

Methods

n/a	Involved in the study	
☒	☐	ChIP-seq
☒	☐	Flow cytometry
☒	☐	MRI-based neuroimaging

Antibodies

Antibodies used	1:100CD71+ anti-human antibody (Mintenyi Biotec, cat # 130-098-779) and 1:500 Alexa Fluor-568 goat anti-mouse secondary antibody (Invitrogen, cat # A-11004) were used to fluorescently stain reticulocytes. 1:100 CD45+ anti-mouse antibody (VWR, clone HI30, cat # 304002-BL) and 1:200 CD41+ integrin alpha 2b antibody (VWR, cat # 10088-996) were used to stain white blood cells and platelets, respectively, in certain experiments, as detailed in supplemented methods.
Validation	All manufacturers have stated that antibody lots are quality control tested by immunofluorescent staining with flow cytometric analysis. Antibody staining was primarily used to detect presence of cells, no conclusions were drawn from antibody intensity.

Eukaryotic cell lines

Policy information about cell lines and Sex and Gender in Research

Cell line source(s)	Cultured human umbilical vein endothelial cells (HUVECs) were used for "endothelialization" experiments (source: Lonza). Primary human blood cell samples were obtained through venipuncture from both male and female subjects. Jurkat cells were obtained from Lonza.
Authentication	Cell lines were not authenticated.
Mycoplasma contamination	Cells lines were not tested for mycoplasma contamination.
Commonly misidentified lines (See ICLAC register)	No commonly misidentified cell lines were used.

Clinical data

Policy information about clinical studies

All manuscripts should comply with the ICMJE guidelines for publication of clinical research and a completed CONSORT checklist must be included with all submissions.

Clinical trial registration	While patient information was used, the study does not meet the criteria of a clinical trial.
Study protocol	While patient information was used, the study does not meet the criteria of a clinical trial.
Data collection	While patient information was used, the study does not meet the criteria of a clinical trial.
Outcomes	While patient information was used, the study does not meet the criteria of a clinical trial.
