# Supplementary material for: iCLOTS: open-source, artificial intelligence-enabled software for analyses of blood cells in microfluidic and microscopy-based assays
Source: Nat Commun. 2023 Aug 18;14:5022. doi: 10.1038/s41467-023-40522-4 (PMC10439163; doi:10.1038/s41467-023-40522-4)
Supplement: Supplementary file 4 — Description of Additional Supplementary Files [file 41467_2023_40522_MOESM4_ESM.pdf]

Description of Additional Supplementary Files

Supplementary Movie 1

Description: Demonstration of iCLOTS capabilities.

Supplementary Movie 2

Description: Demonstration of iCLOTS blood suspension velocity data analysis.
